# Close-Up on Ambulance Service Estimation in Indonesia: Monte Carlo Simulation Study

**DOI:** 10.2196/54240

**Published:** 2024-12-13

**Authors:** Syaribah N Brice, Justin J Boutilier, Geraint Palmer, Paul R Harper, Vincent Knight, Mark Tuson, Daniel Gartner

**Affiliations:** 1 School of Mathematics Cardiff University Cardiff United Kingdom; 2 Telfer School of Management University of Ottawa Ottawa, ON Canada

**Keywords:** emergency medical services, ambulance services, hospital emergency services, Southeast Asian countries, low-and-middle-income countries, EMS, survey

## Abstract

**Background:**

Emergency medical services have a pivotal role in giving timely and appropriate responses to emergency events caused by medical, natural, or human-caused disasters. To provide adequate resources for the emergency services, such as ambulances, it is necessary to understand the demand for such services. In Indonesia, estimates of demand for emergency services cannot be obtained easily due to a lack of published literature or official reports concerning the matter.

**Objective:**

This study aimed to ascertain an estimate of the annual volume of hospital emergency visits and the corresponding demand for ambulance services in the city of Jakarta.

**Methods:**

In this study, we addressed the problem of emergency services demand estimation when aggregated detailed data are not available or are not part of the routine data collection. We used survey data together with the local Office of National Statistics reports and sample data from hospital emergency departments to establish parameter estimation. This involved estimating 4 parameters: the population of each area per period (day and night), the annual per capita hospital emergency visits, the probability of an emergency taking place in each period, and the rate of ambulance need per area. Monte Carlo simulation and naïve methods were used to generate an estimation for the mean ambulance needs per area in Jakarta.

**Results:**

The results estimated that the total annual ambulance need in Jakarta is between 83,000 and 241,000. Assuming the rate of ambulance usage in Jakarta at 9.3%, we estimated the total annual hospital emergency visits in Jakarta at around 0.9-2.6 million. The study also found that the estimation from using the simulation method was smaller than the average (naïve) methods (*P*<.001).

**Conclusions:**

The results provide an estimation of the annual emergency services needed for the city of Jakarta. In the absence of aggregated routinely collected data on emergency medical service usage in Jakarta, our results provide insights into whether the current emergency services, such as ambulances, have been adequately provided.

## Introduction

### Background and Context

Demand for emergency medical services (EMS), in particular ambulance services, has been much discussed in relation to finding factors influencing the change in demand, as well as the benefits of using the ambulance services. Factors influencing the usage of ambulance services can include medical conditions [1], patients’ demographic and socioeconomics [2], environmental factors [3,4], and pandemic events [5,6]. Evidence from the literature also suggests that the use of ambulances reduces prehospital delay [7], which eventually can improve patient-related outcome measures [8].

In Indonesia, natural disasters take place every year including floods, wildfires, earthquakes, landslides, windstorms, tsunamis, and extreme weather conditions [9]. These disasters can affect millions of people including deaths or losses and thousands of injuries. Concerning population health, in 2019, around 19.42% and 14.38% of deaths were caused by stroke and heart disease, respectively [10]. Stroke and ischemic heart disease were among the highest contributors to the years of life lost (6,317 combined) [11]. Trauma cases, including road injuries, contributed to around 4.5% of all deaths [10]. Both natural disasters and ill health can contribute to the demand for ambulance services.

The health care system in developing countries is characterized by scarce resources, including human and financial resources. In Indonesia, health care capacity is far from adequate in many areas and not evenly distributed [12]. The Indonesian prehospital emergency medical services are fragmented and lack standardization, which causes variation in the quality and management of prehospital care delivery. Resources such as ambulances are available, both publicly and privately run. However, lack of funding, coordination, inadequately trained paramedics, and infrastructure are among the issues faced by the emergency ambulance services in Indonesia [13].

Jakarta, the capital of Indonesia, is home to over 11 million people with an area of 664 km^2^ and an annual population growth rate of 0.92% between 2010 and 2020 [14]. In Jakarta, various organizations provide ambulance services. A study found that in Asian countries, ambulance availability ranged from 0.33 to 47 per 100,000 population [15]. The ambulance service includes responding to emergency needs outside the hospitals as well as providing interhospital transportation. Access barriers to ambulance services exist. It is found that many patients, even with life-threatening conditions, travel to hospital emergency departments (EDs) without using appropriate transportation including motorcycles [16]. Factors influencing the patients’ choices for transport are related to medical, socioeconomic, road, and traffic conditions. The congested areas of Jakarta at certain times of the day may suggest the preference for using small and agile transportation such as a motorcycle. The use of public transport or hired cars may be more economical compared with using a private ambulance.

The decisions on ambulance provision in Jakarta are often made without evidence-based or rigorous data analysis. Indeed, it is not easy to know the actual demand in the population. The difficulty in estimating the demand for ambulances is due to several reasons:

1. There is no unifying service that collects data from various ambulance providers. Official reports on health care usage do not cover prehospital emergency services. This could be due to fragmented services, different quality in the data collection process, and lack of resources.
2. Hospitals may collect data on people attending their EDs. However, there are no data available on how the patients arrived at the hospitals. There is no (financial) incentive in collecting such data to support the decision-making process, hence the authority and health care providers do not pay particular attention to collecting data related to transport modes used by the patients.
3. There is no data on the number of people with real emergency conditions who need ambulance services. This issue may not be exclusive to Jakarta. However, factors such as culture and a lack of medical awareness can exacerbate the reluctance to seek help or use an ambulance. For example, it is common in Indonesia for patients with conditions, such as fractures to seek help from alternative treatments, such as from a massage therapist.

### Approaches to Estimating Ambulance Demand

Studies on estimating ambulance demand may have used different approaches depending on the availability of the data. Where data were collected regularly by ambulance providers and available for a long period, a time series forecasting method may be suitable for a short or relatively long period of demand forecasting [17,18]. This method is suitable for anticipating resource requirements needed in a specific time or season. The output is the call volumes for each specific time frame, for example, monthly, daily, or even hourly. This method is built based on past data to mimic the patterns observed in the data with a certain degree of variability as closely as possible. The forecast is deemed relatively accurate when the model output is very close to matching the data. Studies using time series methods will compare several models, using metrics such as mean absolute percentage error, and the best-performing model is to be used to generate the forecast.

An estimation based on different regions can give a granular picture of each region. A spatial forecasting method can be applied in this case where characteristics of each forecasted area are taken into consideration [19]. Machine learning techniques such as multilayer perceptron [20], and artificial neural network [21] can be used in addressing spatiotemporal issues. However, this requires data that capture information on the patient’s location as well as the time. Patients are grouped by region, and characteristics for each region may be used if multivariate models are to be developed. In addition to spatiotemporal issues, different transportation modes can also be considered to build an optimization model for estimating ambulance demand [22].

Estimating the demand for emergency services is a challenging process, particularly when aggregated data are not available. In Indonesia, studies quantifying the demand for ambulance services are scarce. Only one recent paper has attempted to quantify the demand for ED use in Jakarta, [23], using a naïve approach, complemented by regression analyses to fill in missing data, to estimate 2 million ED visits in 2017. We build on the work of [23], with a particular focus on ambulance usage that is missing in that study. Here we differ from that paper by using simulation to estimate variance in demand as well as overall demand level and considering commuters who do not live within the Jakarta city boundaries.

In this study, we aim to estimate the demand for emergency services, including ambulance usage, by considering regional variation at the neighborhood levels within Jakarta. The study serves 2 main purposes: first, to develop a method for estimating annual emergency service usage in the absence of aggregated data, and second, to provide an estimate of the annual usage of hospital emergency services and the need for ambulance services in Jakarta. This report contributes to the existing literature on emergency medical services in low- and middle-income countries such as Indonesia.

## Methods

To estimate the annual emergency service usage, we used population estimates broken down into the smallest administrative areas. The method necessitated computing (1) an estimation of population size for daytime and nighttime; (2) an estimation of the probability of emergency calls coming from each area for daytime and nighttime; (3) an estimation of the rate of ED visits per capita; and (4) an estimation of the rate ambulance needs per area in Jakarta. The details of the 4 elements needed for the estimate will be described in the relevant subsequent section.

### Data Sources

The data-informing parameters used in this study came from various sources: survey data from Brice et al [16], official data obtained from the Indonesian Office for National Statistics as well as the Public Health Office reports, and data from 10 hospital EDs across Jakarta. The key survey in question, [16], collected data such as mode, time, and duration of transport to the hospital, as well as demographic information, for 1,964 patients arriving at five major hospitals in Jakarta in December 2019. We extracted information (from the survey) on the number of patients per unit neighborhood and the methods of transportation to go to the hospitals. The Statistical Office and the Government reports provide an estimation of the characteristics of the population in the region. These include the ratio of male and female population and the population density. We used information reported for the year 2019 (before the COVID-19 pandemic) as subsequent years’ data will be affected by the pandemic. Please note that mainland Jakarta is divided into 5 municipalities. Each municipality administers several districts, and each district is further divided into neighborhoods. There are 267 neighborhoods within the whole Jakarta area. We excluded 6 neighborhoods from the Thousand Island region in northern Jakarta, as data are not always available. Table S1 in Multimedia Appendix 1 summarizes the data gathered from the Indonesian Office for National Statistics and official health reports.

The survey data provide information on whether people used ambulances to go to hospitals. A total of 1964 patients were included in the survey and around 9.3% (183/1964) have used ambulances to go to the hospital’s ED [16]. Table S2 in Multimedia Appendix 2 summarizes the number of ED visits from the survey when data was aggregated by neighborhoods. It shows that around 50% of neighborhoods have used at most 1 ambulance. This is significantly low compared with the number of other transportation used by the patients. We sought information on the number of ED visits in year 2019 from 10 hospitals across Jakarta. Verbal consent was sought from each ED director; however, there was no involvement with their patients or patient-level data. The data were used to estimate the rate per capita of ED used in Jakarta. A summary of this is provided in Table 1 in the parameter estimation section. The hospitals were sampled purposedly to cover all the 5 municipalities in Jakarta.

**Table 1:** Summary of ED^a^ visits from 10 hospitals across Jakarta in 2019.

Measure	Value, n
The sum of ED visits	266,931
Minimum	1,080
First quantile	12,522
Median	24,629
Third quantile	35,730
Maximum	66,899

^a^ED: emergency department.

### Ethical Considerations

The aim of this secondary analysis was covered in the project, titled “Modeling Emergency Medical Services in Indonesia,” approved by the ethics committee from Sumber Waras Hospital, “Rumah Sakit Sumber Waras Komite Etik Penelitian” (001//RSSW/Kom.EP/EC/XI/2019). The element of the study reported in this article used secondary data analysis and adhered to the ethical review and approval processes as required by the Declaration of Helsinki guidelines. The original consent provided by participants included provisions for future use of their data in secondary analyses and all data used were anonymized prior to access to ensure no identifiable information was included. There was no compensation provided to participants.

### Estimation Methods

The study aims to estimate the number of emergency care needs in Jakarta, hence the number of people needing ambulances. The method of estimating the annual number of ED visits using a certain transport method can be found in Boutilier and Chan [22]. However, because of data limitations, we decided to develop a Monte Carlo simulation approach instead of creating a logistic regression model. This Monte Carlo method was used to compare the results with simple naïve models. Naïve (average) models assume that the rate of ambulance use is equal across the neighborhoods, whereas the simulation model accounts for some variation in the neighborhoods. The results will be used to estimate the need for emergency care per neighborhood in Jakarta.

The lack of published hospitals’ data on the number of annual ED visits required us to estimate the following: first, the population per neighborhood for daytime and nighttime. Note that daytime is defined from 6 AM to 5:59 PM, and nighttime is defined from 6 PM to 5:59 AM. This allows us to break the estimation of emergency care needs by the time of the day. Second, the probability that the emergency call happens for each period (ie, daytime or nighttime). Third, the average annual number of ED visits per capita. Fourth, the proportion of ED visits per neighborhood arriving via ambulance. We simplify the description of the estimation model, and its modification as follows:

Let *P_i,j_* be the population size in neighborhood *i* at the period of a day *j*; *ε_j_* be the probability of an emergency happening at the period of a day *j*; *λ_i_* be the expected annual rate of ED visits per capita in neighborhood *i*; and *δ_i_* be the proportion of ED visits from neighborhood *i* that arrived using an ambulance.

The total expected annual number of ED visits in the whole region is the sum of expected annual ED visits in neighborhoods. Mathematically, this is written as:



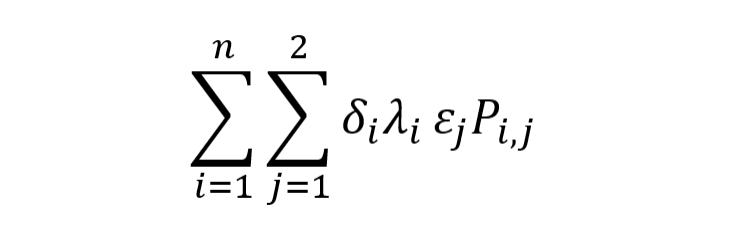



Where *n* is the number of neighborhoods, which in our case is Jakarta (261) and *j* is either daytime or nighttime. The model in reference [22] was modified to include the probability of an emergency happening in each period *j*, here denoted as *ε_j_*.

### Parameter Estimation

#### Step 1: Estimating the Population of Jakarta for Daytime (*P_i,1_*) and Nighttime (*P_i,2_*)

The study only concerns the area of Jakarta within the mainland (ie, excluding the Thousand Island Regency). Jakarta mainland comprises 5 municipalities or cities namely North, Central, West, South, and East. Each city administered several districts, and the total number of districts is 42. Each district is divided into several neighborhoods, totaling 261 neighborhoods within Jakarta.

The Indonesian Office for National Statistics estimated the total population in Jakarta for mid-2019 to be around 10.5 million [24]. Statistics on the number of people commuting in Jakarta in 2019 were estimated in [25], which gave a net total additional population of Jakarta due to commuting is estimated to be around 1.1 million people. It also showed that the number of commuters varies for each city or municipality in Jakarta; the highest additional daytime population is in Central Jakarta (estimated to be over 524 thousand people), followed by South Jakarta (over 418 thousand people). Therefore, it follows that the total population in Jakarta for daytime and nighttime is to be estimated at around 11.6 million and 10.5 million, respectively. The nighttime estimate used the population estimate provided by the Office for National Statistics.

#### Step 2: Estimating the Probability of Calls for Daytime *ε_1_* and Nighttime *ε_2_*

The probability of an emergency happening at period of day *j* (*ε_j_*) is needed since we use the estimation in daytime and nighttime. The probability is estimated from the hospital survey data provided by Brice et al [16], which recorded the time the emergency events happened. We classify the ambulance calls into those that happened in the daytime, between 6 AM to 5:59 PM, and those that happened in the nighttime, 6 PM to 5:59 AM. These were 1228 calls and 736 calls, respectively, giving the proportion of calls happening in daytime and nighttime as 0.625 and 0.375, respectively.

#### Step 3: Estimating the Average Annual Rate of ED Visits Per Capita *λ_i_*

The Public Health Office of DKI Jakarta Province reported that the number of general hospitals in Jakarta is 143 [26]. This does not account for 315 public health clinics, 47 special hospitals, or 19 maternity hospitals. At least around 86 general hospitals have ED. Sample data from 10 hospitals with EDs across Jakarta were sought during this study. The hospitals were purposely sampled to ensure representation from each of the 5 municipalities, with a diverse mix of government, Ministry of Health, defense, and private hospitals. The inclusion of at least one general hospital from each municipality was aimed at ensuring the samples were as representative as possible, allowing us to estimate demand across all EDs. Table 1 summarizes the findings on the number of total ED visits for 2019 from the participating hospitals. Multiplying the median, the first, and the third quantiles to 86 general hospitals, we arrived at a crude estimation for the total annual number of people attending the ED in Jakarta (Table 2).

To arrive at the annual rate of ED visits per capita, we simply divide the estimated total annual ED visits by the total population of Jakarta (in our case 10.5 million). The median number of annual ED visits is 2,118,051 (IQR 1,076,892-3,072,737).

It follows that the crude estimation for the median rate per capita for ED visits is 0.20 (IQR 0.1-0.3; based on a 10.5 million population size and rounded to 1 decimal point). It suggests that the total annual ED visits in Jakarta based on 2019 data is between 1 and 3 million visits. From this, we use the assumption that the rate per capita ED visit is uniform for the whole region.

#### Step 4: Estimating the Yearly Rate for Ambulance Needs Within Jakarta *δ_i_*

We used the survey results of Brice et al [16] on the usage of ambulances per neighborhood. We obtained the count per neighborhood on whether people used ambulances or not to go to the hospitals. Since generally patients are independent of each other, we assumed that people who used ambulances to go to the hospitals were independent of those who used other modes of transportation. To obtain the yearly rate for ambulance need per neighborhood, we used three approaches namely a Monte Carlo simulation [27], a naïve, and a weighted naïve approach. The description of the three approaches can be found in Multimedia Appendix 3. The implementation of the Monte Carlo simulation, together with the naïve and weighted naïve models, and the statistical analyses of the results were done in Python.

**Figure 1:**
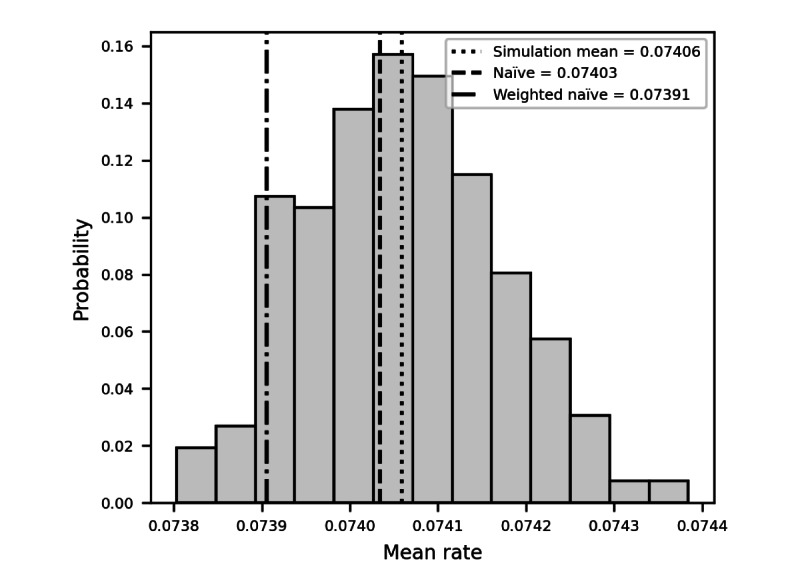
Distribution of the mean rate for ambulance use from simulation results compared with naïve and weighted naïve estimates.

Due to the central limit theorem, the sample means for the rate of ambulance follow a normal distribution, so confidence intervals can be calculated, and parametric *t* tests can be performed comparing the distribution obtained from the Monte Carlo simulation to the naïve and weighted naïve estimated. At the aggregated level, the average sample mean for the rate of using an ambulance was 0.07406 (95% CI 0.07388-0.07424). This is significantly different (*P*<.001 when performing a one-sample *t* test) from both the naïve and weighted naïve estimates, showing that the simulation methodology produces a different estimate from the naïve approaches. Figure 1 illustrates the distribution of the sample means for the rate of ambulance needs.

## Results

Table 2 displays the results from applying naïve, weighted naïve, and Monte Carlo simulation models using different per capita rates of annual ED visits. We calculated the annual ambulance needs for each neighborhood and aggregated the results to give the estimate for the entire region. The computation for the nighttime and the daytime was based on the assumption that the proportions of patients attending the ED during daytime and nighttime are 62.5% and 37.5%, respectively [16].

The results indicate that the annual number of people using ambulances is between around 82,000 and 241,000. Assuming that only 9.3% of people who came to the hospital ED have used an ambulance, we can estimate the total annual ED attendance to be between 0.9 and 2.6 million.

**Table 2:** Estimated annual ED^a^ visits using ambulance split into daytime and nighttime.

Model	Rate per capita ED visits	Rate ambulance use	Nighttime est.	Daytime est.	Total ambulance needs	Total annual emergency care need
Naïve	0.10	0.07406	29,203	53,871	83,074	893,269
Weighted naïve	0.10	0.07403	29,161	53,784	82,945	891,882
Simulation	0.10	0.07391	29,221	53,905	83,126	893,828
Naïve	0.20	0.07406	58,402	107,751	166,153	1,786,591
Weighted naïve	0.20	0.07403	58,295	107,560	165,855	1,783,387
Simulation	0.20	0.07391	58,428	107,794	166,222	1,787,333
Naïve	0.29	0.07406	84,674	156,237	240,911	2,590,441
Weighted naïve	0.29	0.07403	84,532	155,951	240,483	2,585,839
Simulation	0.29	0.07391	84,705	156,295	241,000	2,591,398

^a^ED: emergency department.

**Figure 2:**
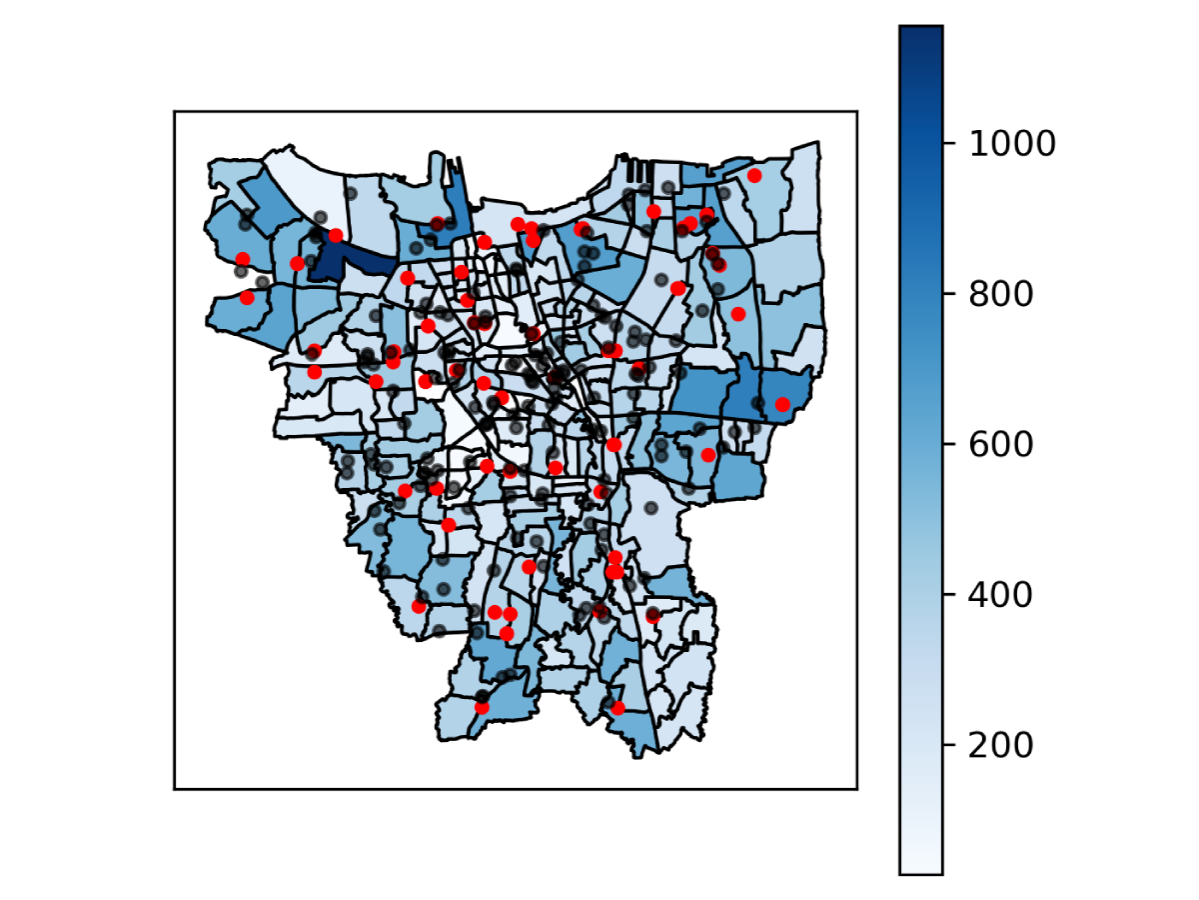
Distribution for annual ambulance demand and location for ambulance posts (red) and hospitals (black).

Figure 2 depicts the distribution of annual ambulance needs estimated from simulation results across the city of Jakarta. The red dots represent the geographic locations of the ambulance posts. The black dots represent the geographical location of hospitals. The vast majority of neighborhoods have no ambulance. While in a small number of neighborhoods, ambulance posts can be quite close to one another. The total number of hospitals captured is 168 with varying classifications including general and specialist hospitals, belonging to the government as well as privately funded. Hospitals with EDs are mainly located around the central region of Jakarta.

## Discussion

### Principal Findings

The current study estimated the annual demand for emergency services in Jakarta to be between 0.9 and 2.6 million and for ambulance services to be between 83,000 and 241,000. The estimation derived through the simulation method proved to be slightly higher than the results obtained from using the conventional averaging methods (*P*<.001). This variation could be attributed to the fact that average or naïve methods, by definition, may be affected by exceptionally small or large values within the data. Moreover, the naïve methods assumed that the rate of demand for emergency services was the same across the entire city of Jakarta, irrespective of variations in population characteristics within its neighborhoods, whereas the simulation method generated random sampling depending on the distribution of the sample values. Hence, the resultant estimates varied from one neighborhood to another. Considering the small differences in the results, the use of average models remains reasonable in this case for two reasons. First, the neighborhoods in Jakarta exhibit similar population characteristics and the Office for National Statistics in Jakarta only produces reports at aggregate levels (municipalities). Second, while patients’ characteristics and medical conditions may affect the increased usage of emergency services, regional differences such as density do not [28].

The estimated demand of 2.6 million annual ED visits was based on 2019 data. A previous attempt used a survey method to estimate 2 million ED visits in Jakarta for 2017 [23], and we now estimate a higher level of demand. This could be for two reasons: first, an increase in the population of Jakarta, including an aging population [29], would result in higher demand compared with 2017; and second, our study considers daytime commuters that contribute to the demand, where the previous study does not. Like any estimation study, it cannot provide an exact measure of demand. Indeed, the true demand for health care services can never be known, even with routine data collection. Nevertheless, our study suggests two key points: first, there is a pressing need for the Indonesian government, particularly the local government in Jakarta, to collect regional and national data on emergency services usage. These data can be instrumental in studying the capacity and demand for comprehensive emergency medical services including prehospital, hospital, and ambulance services, as many capacity and planning models on the operational and strategic levels require detailed demand and variation data, while tactical planning requires a good estimate of current demand to efficiently and effectively plan for the future. Ensuring well-structured and adequately supported emergency medical services at all levels, from community to national, is essential for efficiently serving the Indonesian population [30]. Second, the study’s estimated demand volume suggests a significant need for ambulance services. While government-owned ambulances in Jakarta are provided to the population free of charge, a study revealed that less than 10% of individuals seeking hospital emergency care arrived via ambulance [16]. This warrants an investigation into whether the existing ambulance capacity is sufficient to meet the needs of Jakarta’s population, which exceeds 11 million people.

### Comparison With Prior Work

Our study provided the first estimate of emergency services usage in Jakarta using the population estimation method considering spatial and time variation. It followed the method used by Boutilier and Chan [22] with a modification. Our formulation included the probability of emergency events happening in the nighttime and daytime. In addition, we used a Monte Carlo simulation due to limited data instead of logistic regression. When data are sufficiently large and combined with multiple variables, a machine-learning technique can be developed as implemented in Boutilier and Chan [22].

The accuracy of the simulation results depends on the underlying distribution used to generate the samples. Our samples, on the emergency medical services used, came from a cross-sectional study [16] that surveyed the usage of EMS in one month. Due to a lack of aggregated data on the use of EMS in Jakarta, the results from the current study cannot be validated.

In the absence of routinely collected data, the current study used various data sources in conjunction with survey data to parameterize the models. Previous research on health care services demand has adopted diverse methodologies to generate more precise estimations. For example, in the palliative care demand, routinely collected data can be used in an estimation model to include deaths as well as the prevalence of the disease [31]. Simple linear projection modeling can be used, complementing expert opinions and online census data [32] to project health care needs. By combining different data sources, such as hospital and census data, more detailed results can be achieved. The analyses can include the regional variation in the risk of emergency related to patients’ characteristics such as age, sex, comorbidities, and socioeconomics (see an example in [33]). In Jakarta, a survey method involving many hospital EDs was used to estimate annual ED visits [23].

### Limitations

Finally, the study has limitations, as mentioned above, primarily related to the absence of the routinely collected aggregated data. Other limitations are related to the construction of the model. The model did not incorporate the different transportation modes used by patients when attending the hospital EDs. Future studies could consider incorporating these modes to obtain different estimations based on transportation types. Furthermore, combining the survey data and the hospital data could be explored to investigate whether patients’ choice of transportation during emergencies affects patient outcomes.

### Conclusions

In this paper, we used Monte Carlo Simulation methods to estimate the annual demand for EDs and ambulance usage needed for the city of Jakarta, by extrapolating and sampling from survey data, and considering commuter behaviors that affect the daytime-nighttime population cycle of the city. This methodology includes getting an estimate of the distribution of the demand, and therefore its variability, which can be useful in downstream capacity and planning modeling. We compared the results obtained with a naïve forecasting approach. The demand estimation is evaluated on different metrics and levels of detail and provides a breakdown into the smallest administrative areas (neighborhoods). Considering the limited resources of ambulances and the absence of routinely collected data on EMS usage in Jakarta, the results can be used to investigate further whether the current emergency services, such as ambulances, have been equally and adequately provided.

## Appendix

Multimedia Appendix 1 Table S1. Population characteristics by neighbourhoods.

Multimedia Appendix 2 Table S2. Summary of transport used per neighbourhood to go to hospitals from survey data.

Multimedia Appendix 3 Description of naïve, weighted naïve and Monte Carlo approaches used in the study.

## Abbreviations

ED: emergency department
EMS: emergency medical services

## Data Availability

All data generated or analyzed during this study and their sources were mentioned in this published manuscript.
