# Supplementary material for: Close-Up on Ambulance Service Estimation in Indonesia: Monte Carlo Simulation Study
Source: Interact J Med Res. 2024 Dec 13;13:e54240. doi: 10.2196/54240 (PMC11681287; doi:10.2196/54240)
Supplement: Multimedia Appendix 1 [file ijmr_v13i1e54240_app1.pdf]

Appendix 1

*Table S1. Population characteristics by neighbourhoods*

| Characteristic | Jakarta All neighborhoods | Minimum | Mean | Maximum |
|---|---|---|---|---|
| Population size | 10,514,799 | 3,191 | 40,287 | 154,813 |
| Area (km2) | 662.4 | 0.3 | 2.5 | 13.1 |
| Population density | 15,873 | 1,227 | 23,129.8 | 136,258 |
| Ratio male/Female 65+ | 0.90 | 0.80 | 0.90 | 0.96 |
| Ratio male/female under 15 | 1.04 | 1.03 | 1.04 | 1.08 |
| Ratio male/female married | 1.02 | 1.00 | 1.01 | 1.03 |
| Ratio male/female literacy rate | 1.00 | 1.00 | 1.00 | 1.00 |
| Ratio male/female having a senior high certificate and or above | 1.10 | 1.06 | 1.11 | 1.13 |
| Ratio male/female ratio used health insurance to access healthcare | 1.01 | 0.88 | 1.00 | 1.08 |
