# Supplementary material for: Close-Up on Ambulance Service Estimation in Indonesia: Monte Carlo Simulation Study
Source: Interact J Med Res. 2024 Dec 13;13:e54240. doi: 10.2196/54240 (PMC11681287; doi:10.2196/54240)
Supplement: Multimedia Appendix 2 [file ijmr_v13i1e54240_app2.pdf]

*Table S2. Summary of transport used per neighbourhood to go to hospitals from survey data.*

| Variables | Min | Q1 | Median | Q3 | Max |
|---|---|---|---|---|---|
| Ambulance usage | 0 | 0 | 1 | 2.8 | 10 |
| Other vehicles | 0 | 3 | 12 | 23.8 | 178 |
| Total ED visits | 0 | 3 | 13 | 25 | 188 |
