# Supplementary material for: Close-Up on Ambulance Service Estimation in Indonesia: Monte Carlo Simulation Study
Source: Interact J Med Res. 2024 Dec 13;13:e54240. doi: 10.2196/54240 (PMC11681287; doi:10.2196/54240)
Supplement: Multimedia Appendix 3 [file ijmr_v13i1e54240_app3.pdf]

Appendix 3

Description of naïve, weighted naïve and Monte Carlo approaches used in the study.

The naive approach calculated the simple mean for people using ambulances as a proportion of the total number of people who came to the hospital ED in a year. The rate of using ambulances can be described as:

$$\delta = \frac{\sum_{i=1}^{n} x_i}{\sum_{i=1}^{n} (y_i + x_i)}$$

Where x_i and y_i are the count of people using and not using ambulances in area i respectively and *n* is the number of neighbourhoods.

The weighted naïve approach calculates the weighted mean for people who used ambulances and people who did not use ambulances. The rate of ambulance needs is the weighted proportion of those people who used ambulances over the total people who came to the hospital in a year based on weighted naïve estimates. The description for the weighted naïve can be simplified as:

$$\delta = \frac{\sum_{i=1}^{n} (x_i * z_i)}{\sum_{i=1}^{n} (y_i * z_i) + \sum_{i=1}^{n} (x_i * z_i)}$$

Where x_i , y_i and *n* are as described in the naïve model; $z_i = x_i + y_i$, i.e., the total number of people who went to the hospital in the area *i*.

A Monte Carlo simulation is a technique known for generating a large number of random samples from a defined probability distribution (see [27] for a detailed technical description). The use of Monte Carlo simulation in this case can be justified for two reasons. The first is that the demand for ambulances in each area is independent of other areas based on our

aforementioned assumption of independent patients. The second is that we can determine the distributions for the number of people using ambulances and the number of people not using ambulances in the following way: study [16] gives an empirical distribution of ED demand from each neighbourhood, with missing values as it surveyed only a sample of EDs. We can extrapolate this to all neighbourhoods by randomly sampling from this empirical distribution, as we assume that the neighbourhoods are uniform and independent of one another. The idea of using Monte Carlo simulation is to generate large samples for the number of people using ambulances and not using ambulances for each area. To generate a sample demand per neighbourhood, the simulation was run 500 times with 1000 samples generated at each run. The sample mean was computed to give the estimate for the number of people who use ambulances and do not use ambulances in the area. The rate of using ambulances was calculated as the proportion of people using ambulances of the total people. More precisely, the rate for ambulance needs/use in each area *i* (δ_i) can be described as:

$$\delta_i = \frac{1}{m} \frac{\sum_{j=1}^{l} x_j}{\sum_{j=1}^{l} x_j + \sum_{j=1}^{l} y_j}$$

Where x_j is the mean number of people who use ambulances, y_j is the mean number of people who do not use the ambulances generated at random sampling *j*, *l* is the sample size (1000), and *m* is the number of simulation runs (500). This technique inherently approximates the distribution of the demand rate, a useful measure not estimated in the naïve approaches.
